# Differences in clinical characteristics and prognosis between breast neuroendocrine carcinoma and breast invasive ductal carcinoma: A multicentre population‐based study from China

**DOI:** 10.1002/cam4.5819

**Published:** 2023-03-23

**Authors:** Ding‐yuan Wang, Shuo‐feng Li, Song‐lin Gao, Peng Yuan, Bai‐lin Zhang

**Affiliations:** ^1^ Department of Breast Surgery, National Cancer Center/National Clinical Research Center for Cancer/Cancer Hospital Chinese Academy of Medical Sciences and Peking Union Medical College 100021 Beijing China; ^2^ Department of Colorectal Surgery, National Cancer Center/National Clinical Research Center for Cancer/Cancer Hospital Chinese Academy of Medical Sciences and Peking Union Medical College 100021 Beijing China; ^3^ Department of VIP Medical Services, National Cancer Center/National Clinical Research Center for Cancer/Cancer Hospital Chinese Academy of Medical Sciences and Peking Union Medical College 100021 Beijing China

**Keywords:** breast carcinoma, invasive ductal carcinoma, neuroendocrine carcinoma, prognosis

## Abstract

**Aim:**

We constructed a multicentre cohort in China to analyse the differences in clinical characteristics, treatment strategies and prognoses between breast neuroendocrine carcinoma (NEC) and invasive ductal carcinoma (IDC) of the breast.

**Methods:**

All patients with early‐stage breast cancer who attended three hospitals in Beijing from 2000 to 2018 were included in the study. We used propensity score matching to make a 1:3 match between NEC and IDC.

**Results:**

After propensity score matching, 153 patients with IDC and 51 patients with NEC were analysed. Multivariate Cox regression showed that compared to patients with IDC, patients with NEC had a worse disease‐free survival (HR = 2.94, 95% CI: 1.69–5.12, *p* < 0.001).

**Conclusion:**

NEC patients have a worse disease‐free survival than IDC patients.

## Graphical abstract

Neuroendocrine carcinoma (NEC) of the breast is a very rare pathological type with few studies illustrating the prognosis compared with invasive ductal carcinoma (IDC). Our team used propensity score matching to make a 1:3 match between NEC and IDC. Thus, 153 patients with IDC and 51 patients with NEC were analysed. Compared to patients with IDC, patients with NEC had a worse disease‐free survival (HR = 2.94, 95% CI: 1.69–5.12, *p* < 0.001).

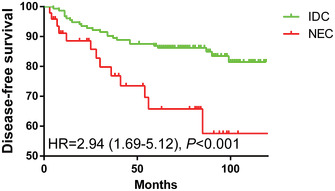

## 1 INTRODUCTION

Neuroendocrine carcinoma (NEC) of the breast is a very rare pathological type, accounting for approximately 1% of neuroendocrine tumours and only 0.1% of invasive breast cancers.^1^, ^2^, ^3^ Treatment modalities for breast NEC are difficult to study, as the prognostic features of breast NEC have not been elucidated by a majority of clinicians. The prognostic relevance of neuroendocrine differentiation in breast carcinoma is still debated because several studies have been published with mixed results.^2^, ^4^, ^5^ These conflicting results might be explained by the limited number of cases reported in each series. Our team constructed a multicentre cohort to analyse the differences in clinical characteristics, treatment strategies and prognosis between NEC and the most common pathologic type of breast cancer, invasive ductal carcinoma (IDC) of the breast.

## 2 METHODS

This retrospective study was performed in line with the principles of the Declaration of Helsinki, and it was reviewed and approved by the authorities of the Ethics Committee of National Cancer Centre/National Clinical Research Centre for Cancer/Cancer Hospital, Chinese Academy of Medical Sciences and Peking Union Medical College (22/035‐3236 and 20/272‐2468). Patient consent was not required for this study, as there were no risks anticipated to the participants of the study. All data were stripped of any patient identifiers, and all data will be reported in the aggregate.

We collected breast cancer patients who visited the Cancer Hospital of the Chinese Academy of Medical Sciences, Beijing Cancer Hospital and Beijing Hospital between 2000 and 2018. Only one month from March to December of a year was selected randomly and all patients in that month were included as representatives of that particular year. Patients in the subsequent month of the next year were recruited. If the number of cases in the selected month could not meet the requirement, more cases from the subsequent month were included until the total number in that hospital met the requirement. The exclusion criteria were as follows: (i) other pathological types; (ii) de novo stage IV breast cancer; (iii) no surgical treatment; and (iv) missing survival information. Patient age, laterality, stage, grade and molecular subtype were obtained from the medical records. All information was collected from a routine medical records system using a self‐designed case report form (CRF). Additional details are provided in another study by our team.^6^

We used propensity score matching to make a 1:3 match between NEC and IDC. Differences in the distribution of each variable between groups were analysed using chi‐square tests or Fisher's exact test. Survival analysis was performed using univariate and multivariate Cox regression. Overall survival (OS) was defined as the time from the date of diagnosis to the date of death due to any cause or the last follow‐up. Disease‐free survival (DFS) was defined as the time from the date of radical surgery to the date of death due to any cause, recurrence/metastasis or the last follow‐up. All analyses were performed in R 4.0.1. GraphPad Prism 6 was used to plot survival curves. Two‐sided tests were used in all papers. A *p* value less than 0.05 was considered statistically significant.

## 3 RESULTS

A total of 3042 patients with IDC and 51 patients with NEC were included in the study. The median follow‐up was 75 months for the full cohort and 77 and 40 months for IDC and NEC, respectively. A total of 718 (23.60%) of the IDC patients were older than 60 years, and 28 (54.90%) of the NEC patients were older than 60 years, with statistically significant differences (*p* < 0.001). A total of 1225 (40.27%) patients with IDC had Ki‐67 higher than 15%, and 28 (54.90%) patients with NEC had Ki‐67 higher than 15%, and the difference between the two was statistically significant (*p* = 0.006).

After propensity score matching, 153 patients with IDC and 51 patients with NEC were analysed. There were no significant differences in age, laterality, stage, grade, molecular subtype or treatment strategy between IDC and NEC (Table 1).

**TABLE 1:** Clinical characteristics and treatment strategies in breast neuroendocrine carcinoma and invasive ductal carcinoma.

	Before matching			After matching		
	IDC, No.(%)^a^	NEC, No.(%)^b^	*p*	IDC, No.(%)	NEC, No.(%)	*p*
Overall	3042	51		153	51	
Age			<0.001***			0.518
<60	2324 (76.40)	23 (45.10)		79 (51.63)	23 (45.10)	
≥60	718 (23.60)	28 (54.90)		74 (48.37)	28 (54.90)	
Laterality			<0.001***			0.151
Left	1509 (49.61)	32 (62.75)		84 (54.90)	32 (62.75)	
Right	1499 (49.28)	16 (31.37)		67 (43.79)	16 (31.37)	
Bilateral	33 (1.08)	2 (3.92)		1 (0.65)	2 (3.92)	
Unknown	1 (0.03)	1 (1.96)		1 (0.65)	1 (1.96)	
TNM stage			0.210			0.870
I	925 (30.41)	13 (25.49)		31 (20.26)	13 (25.49)	
II	1597 (52.50)	32 (62.75)		105 (68.63)	32 (62.75)	
III	416 (13.68)	3 (5.88)		9 (5.88)	3 (5.88)	
Unknown	104 (3.42)	3 (5.88)		8 (5.23)	3 (5.88)	
Grade			0.400			0.541
G1 or G2	2133 (70.12)	35 (68.63)		98 (64.05)	35 (68.63)	
G3	663 (21.79)	14 (27.45)		52 (33.99)	14 (27.45)	
Unknown	246 (8.09)	2 (3.92)		3 (1.96)	2 (3.92)	
ER^c^			0.175			1.000
Negative	858 (28.21)	9 (17.65)		28 (18.30)	9 (17.65)	
Positive	2152 (70.74)	42 (82.35)		125 (81.70)	42 (82.35)	
Unknown	32 (1.05)	0 (0.00)		0 (0.00)	0 (0.00)	
PR^d^			0.222			1.000
Negative	955 (31.39)	11 (21.57)		33 (21.57)	11 (21.57)	
Positive	2053 (67.49)	40 (78.43)		120 (78.43)	40 (78.43)	
Unknown	34 (1.12)	0 (0.00)		0 (0.00)	0 (0.00)	
HER2^e^			0.087			0.241
Negative	1953 (64.20)	34 (66.67)		114 (74.51)	34 (66.67)	
Positive	618 (20.32)	5 (9.80)		6 (3.92)	5 (9.80)	
Unknown	471 (15.48)	12 (23.53)		33 (21.57)	12 (23.53)	
Ki‐67 levels			0.006**			0.983
<15%	674 (22.16)	15 (29.41)		43 (28.10)	15 (29.41)	
≥15%	1225 (40.27)	28 (54.90)		86 (56.21)	28 (54.90)	
Unknown	1143 (37.57)	8 (15.69)		24 (15.69)	8 (15.69)	
Chemotherapy			0.002**			0.344
No	628 (20.64)	20 (39.22)		47 (30.72)	20 (39.22)	
Yes	2414 (79.36)	31 (60.78)		106 (69.28)	31 (60.78)	
Radiotherapy			0.415			1.000
No	2199 (72.29)	40 (78.43)		119 (77.78)	40 (78.43)	
Yes	843 (27.71)	11 (21.57)		34 (22.22)	11 (21.57)	
Endocrine therapy			0.001***			1.000
No	1600 (52.60)	14 (27.45)		42 (27.45)	14 (27.45)	
Yes	1442 (47.40)	37 (72.55)		111 (72.55)	37 (72.55)	
CgA^f^						
Negative		7 (13.73)			7 (13.73)	
Positive		31 (60.78)			31 (60.78)	
Unknown		13 (25.49)			13 (25.49)	
SyN^g^						
Negative		7 (13.73)			7 (13.73)	
Positive		28 (54.90)			28 (54.90)	
Unknown		16 (31.37)			16 (31.37)	

^a^ IDC, invasive ductal carcinoma.

^b^ NEC, neuroendocrine carcinoma.

^c^ ER, oestrogen receptor.

^d^ PR, progesterone receptor.

^e^ HER2, human epidermal growth factor receptor‐2.

^f^ CgA, chromogranin A.

^g^ SyN, synaptophysin.

*** *p* ≤ 0.001

** 0.001 < *p* ≤ 0.01.

**FIGURE 1:**
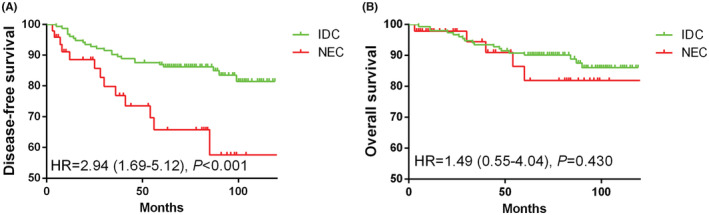
(A) Neuroendocrine carcinomas of the breast have a shorter disease‐free survival than invasive ductal carcinomas. (B) There was no significant difference in on overall survival between patients with NEC and patients with IDC. HR, hazard ratio; IDC, invasive ductal carcinoma; NEC, neuroendocrine carcinoma.

The analysis of survival on DFS is shown in Table S1. For the overall population, univariate Cox regression showed a statistically significant effect of pathological type, stage, grade, oestrogen receptor (ER), progesterone receptor (PR), Ki‐67, chemotherapy and radiotherapy on DFS. Multivariate Cox regression of these variables selected for significance showed that NEC had a worse DFS than IDC (hazard ratio (HR) = 3.24, 95% CI: 1.86–5.65, *p* < 0.001). After propensity score matching, univariate Cox regression showed that NEC had a worse DFS than IDC (HR = 2.77, 95% CI: 1.40–5.48, *p* = 0.003). After adjusting for grade, human epidermal growth factor receptor‐2 (HER2) and Ki‐67, multivariate Cox regression showed that compared to IDC, NEC had a worse DFS (HR = 2.94, 95% CI: 1.69–5.12, *p* < 0.001) (Figure 1A). There was no significant difference on OS between NEC and IDC before and after propensity score matching (Figure 1B).

## 4 DISCUSSION

The incidence of NEC has been reported to be less than 0.1%.^2^ The rarity of NEC makes it challenging to research. Neuroendocrine differentiation may make breast cancer cells more similar to the luminal type.^7^, ^8^ The validity of multigene models such as Oncotype DX in classifying the risk of luminal‐type breast cancer has been confirmed by numerous studies.^9^, ^10^, ^11^ Future studies may be implemented to verify the value of Oncotype DX in NEC to provide more convenient and accurate prognostic stratification for NEC patients.

The prognosis of breast neuroendocrine neoplasms remains very confusing, mostly due to their conceptual inconsistency. Breast cancers with neuroendocrine differentiation can be divided into three categories: NEC, neuroendocrine tumours and invasive breast cancer with neuroendocrine morphology. Patients with neuroendocrine morphology were considered to have a prognosis pattern that is similar,^12^ even better,^13^ compared to IDC. Several studies have confirmed that NEC has a higher rate of local recurrence, a higher rate of lymph node metastasis, and worse survival than IDC.^14^, ^15^ Considering that clinical variables such as age at initial diagnosis, molecular subtype and tumour stage may be potential confounders in survival analysis, coupled with the fact that pathological grade and Ki‐67 are prognostic factors for NEC,^6^ our team obtained a similar conclusion after eliminating differences in clinical characteristics between IDC and NEC cohorts by propensity score matching that NEC had worse DFS than IDC. Therefore, we believe that the type of breast neuroendocrine neoplasms should be clarified during survival analysis to give readers a clearer elucidation.

According to the National Comprehensive Cancer Network (NCCN) Clinical Practice Guideline, the treatment protocols for breast cancer with neuroendocrine differentiation and IDC are of the same standard.^16^ It is evident that more precise and detailed guidelines, such as different strategies for different kinds of neuroendocrine differentiation, need to be explored. According to the analysis in this paper, the worse DFS of NEC implies a higher risk of recurrence or metastasis after radical surgery. Therefore, it may be necessary to implement active post‐operative monitoring and rigorous observation for patients diagnosed with NEC.

Retrospective studies have shown that adjuvant chemotherapy (etoposide + platinum) could benefit the survival of patients with gastrointestinal NEC.^17^, ^18^ Nevertheless, the existing evidence is inadequate to support the use of adjuvant chemotherapy as a viable treatment option for patients with breast NEC. To aid oncologists in making informed decisions regarding adjuvant treatments for their patients, conducting retrospective studies with larger sample sizes based on hospital populations could prove to be useful.

Our study has two significant advantages. First, we balanced the baseline information of IDC and NEC using propensity score matching, making the prognostic difference between IDC and NEC not influenced by potential confounders. Second, compared to a single‐centre study, this multicentre study would have a better representation of patients. However, our study has several potential limitations. First, all patients were included in tertiary care hospitals in Beijing, where patients' conditions may be more complex. Second, although this is a multicentre study, the sample size of this study remains undeniably small due to the rarity of NEC. We believe that NEC is a tumour type that is difficult to treat and worth studying. Therefore, multicentre studies with larger sample sizes are urgently needed. Finally, genomic information was missing in this paper to provide more insight into the reasons why NEC is more likely to relapse. Our team plans to perform whole‐exome sequencing of formalin‐fixed paraffin‐embedded specimens of NEC in the future to develop a more accurate multigene model.

## 5 CONCLUSION

Neuroendocrine carcinoma patients are older and have a worse DFS than IDC patients. Furthermore, the TNM stage, grade, ER and PR status of IDC and NEC exhibit resemblance.

## AUTHOR CONTRIBUTIONS

**Ding‐yuan Wang:** Data curation (equal); investigation (equal); methodology (equal); software (equal); validation (equal); visualization (equal); writing – original draft (equal); writing – review and editing (equal). **Shuofeng Li:** Data curation (equal); formal analysis (equal); methodology (equal); resources (equal); writing – original draft (equal); writing – review and editing (equal). **Song‐lin Gao:** Data curation (equal); formal analysis (equal); investigation (equal); methodology (equal); software (equal); writing – original draft (equal). **Peng Yuan:** Conceptualization (equal); funding acquisition (equal); project administration (equal); resources (equal); supervision (equal). **Bai‐lin Zhang**: Conceptualization (equal); funding acquisition (equal); project administration (lead); resources (equal); supervision (lead).

## FUNDING INFORMATION

This work was supported by Beijing Municipal Natural Science Foundation (7222145 and 7222150).

## CONFLICT OF INTEREST STATEMENT

Not applicable.

## ETHICS STATEMENT

This study was reviewed and approved by the authorities of the Ethics Committee of National Cancer Centre/National Clinical Research Centre for Cancer/Cancer Hospital, Chinese Academy of Medical Sciences and Peking Union Medical College (22/035–3236 and 20/272‐2468).

## Supporting information


Table S1

## Data Availability

The datasets generated and analysed are available from the corresponding author on reasonable request. In addition, data of NEC of breast diagnosed in Beijing Hospital were published online^19^.
