# Supplementary material for: Differences in clinical characteristics and prognosis between breast neuroendocrine carcinoma and breast invasive ductal carcinoma: A multicentre population‐based study from China
Source: Cancer Med. 2023 Mar 23;12(8):9644–9. doi: 10.1002/cam4.5819 (PMC10166936; doi:10.1002/cam4.5819)
Supplement: Supplementary file 1 — Table S1 [file CAM4-12-9644-s001.docx]

**Supplementary Table 1** Survival analysis on disease-free survival

|  | Before matching | | | | After matching | | | |
| --- | --- | --- | --- | --- | --- | --- | --- | --- |
|  | Univariable analysis | | Multivariable analysis | | Univariable analysis | | Multivariable analysis | |
|  | HR (95% CI) ^a^ | *p* | HR (95% CI) | *p* | HR (95% CI) | *p* | HR (95% CI) | *p* |
| Histological type |  |  |  |  |  |  |  |  |
| IDC ^b^ | Reference |  | Reference |  | Reference |  | Reference |  |
| NEC ^c^ | 2.57 (1.48-4.46) | 0.001 | 3.24 (1.86-5.65) | <0.001 | 2.77 (1.40-5.48) | 0.003 | 2.94 (1.69-5.12) | <0.001 |
| Age |  |  |  |  |  |  |  |  |
| ＜60 | Reference |  |  |  | Reference |  |  |  |
| ≥60 | 1.20 (0.99-1.46) | 0.063 |  |  | 0.90 (0.47-1.73) | 0.757 |  |  |
| Laterality |  |  |  |  |  |  |  |  |
| Left | Reference |  |  |  | Reference |  |  |  |
| Right | 0.92 (0.77-1.10) | 0.358 |  |  | 0.93 (0.48-1.80) | 0.819 |  |  |
| Bilateral | 1.54 (0.79-2.99) | 0.205 |  |  | NA |  |  |  |
| TNM stage |  |  |  |  |  |  |  |  |
| I | Reference |  | Reference |  | Reference |  |  |  |
| II | 2.44 (1.87-3.19) | <0.001 | 2.35 (1.80-3.09) | <0.001 | 1.71 (0.66-4.43) | 0.272 |  |  |
| III | 6.46 (4.86-8.59) | <0.001 | 4.88 (3.63-6.57) | <0.001 | 3.19 (0.86-11.88) | 0.084 |  |  |
| Tumour grade |  |  |  |  |  |  |  |  |
| G1 or G2 | Reference |  | Reference |  | Reference |  | Reference |  |
| G3 | 1.89 (1.56-2.29) | <0.001 | 1.40 (1.14-1.72) | 0.001 | 2.17 (1.14-4.13) | 0.019 | 1.72 (1.42-2.10) | <0.001 |
| ER ^d^ |  |  |  |  |  |  |  |  |
| Negative | Reference |  | Reference |  | Reference |  |  |  |
| Positive | 0.62 (0.52-0.75) | <0.001 | 0.84 (0.66-1.07) | 0.162 | 0.71 (0.33-1.56) | 0.400 |  |  |
| PR ^e^ |  |  |  |  |  |  |  |  |
| Negative | Reference |  | Reference |  | Reference |  |  |  |
| Positive | 0.69 (0.57-0.82) | <0.001 | 0.85 (0.67-1.08) | 0.178 | 0.94 (0.43-2.06) | 0.884 |  |  |
| HER2 ^f^ |  |  |  |  |  |  |  |  |
| Negative | Reference |  |  |  | Reference |  | Reference |  |
| Positive | 1.18 (0.95-1.46) | 0.127 |  |  | 3.02 (1.05-8.68) | 0.040 | 1.08 (0.87-1.33) | 0.497 |
| Ki-67 levels |  |  |  |  |  |  |  |  |
| <55% | Reference |  | Reference |  | Reference |  | Reference |  |
| ≥55% | 1.71 (1.30-2.25) | <0.001 | 1.40 (1.06-1.86) | 0.018 | 3.43 (1.19-9.90) | 0.022 | 1.59 (1.21-2.10) | 0.001 |
| Chemotherapy |  |  |  |  |  |  |  |  |
| No | Reference |  | Reference |  | Reference |  |  |  |
| Yes | 2.08 (1.59-2.72) | <0.001 | 1.24 (0.94-1.64) | 0.133 | 1.35 (0.65-2.78) | 0.422 |  |  |
| Radiotherapy |  |  |  |  |  |  |  |  |
| No | Reference |  | Reference |  | Reference |  |  |  |
| Yes | 2.14 (1.80-2.55) | <0.001 | 1.58 (1.29-1.94) | <0.001 | 1.90 (0.95-3.79) | 0.070 |  |  |
| Endocrine therapy |  |  |  |  |  |  |  |  |
| No | Reference |  |  |  | Reference |  |  |  |
| Yes | 1.12 (0.94-1.33) | 0.198 |  |  | 0.86 (0.42-1.73) | 0.664 |  |  |

^a^ HR (95% CI), hazard ratio and 95% confidence interval

^b^ IDC, invasive ductal carcinoma

^c^ NEC, neuroendocrine carcinoma

^d^ ER, oestrogen receptor

^e^ PR, progesterone receptor

^f^ HER2, human epidermal growth factor receptor-2
